# Mycobacterial Dihydrofolate Reductase Inhibitors Identified Using Chemogenomic Methods and *In Vitro* Validation

**DOI:** 10.1371/journal.pone.0121492

**Published:** 2015-03-23

**Authors:** Grace Mugumbate, Katherine A. Abrahams, Jonathan A. G. Cox, George Papadatos, Gerard van Westen, Joël Lelièvre, Szymon T. Calus, Nicholas J. Loman, Lluis Ballell, David Barros, John P. Overington, Gurdyal S. Besra

**Affiliations:** 1 European Molecular Biology Laboratory, European Bioinformatics Institute (EMBL-EBI), Wellcome Trust Genome Campus, Hinxton, Cambridge, United Kingdom; 2 Institute of Microbiology and Infection (IMI), School of Biosciences, University of Birmingham, Edgbaston, Birmingham, United Kingdom; 3 Diseases of the Developing World, GlaxoSmithKline, Severo Ochoa 2, 28760 Tres Cantos, Madrid, Spain; University of Delhi, INDIA

## Abstract

The lack of success in target-based screening approaches to the discovery of antibacterial agents has led to reemergence of phenotypic screening as a successful approach of identifying bioactive, antibacterial compounds. A challenge though with this route is then to identify the molecular target(s) and mechanism of action of the hits. This target identification, or deorphanization step, is often essential in further optimization and validation studies. Direct experimental identification of the molecular target of a screening hit is often complex, precisely because the properties and specificity of the hit are not yet optimized against that target, and so many false positives are often obtained. An alternative is to use computational, predictive, approaches to hypothesize a mechanism of action, which can then be validated in a more directed and efficient manner. Specifically here we present experimental validation of an *in silico* prediction from a large-scale screen performed against *Mycobacterium tuberculosis* (*Mtb*), the causative agent of tuberculosis. The **two** potent anti-tubercular compounds studied in this case, belonging to the tetrahydro-1,3,5-triazin-2-amine (THT) family, were predicted and confirmed to be an inhibitor of dihydrofolate reductase (DHFR), a known essential *Mtb* gene, and already clinically validated as a drug target. Given the large number of similar screening data sets shared amongst the community, this *in vitro* validation of these target predictions gives weight to computational approaches to establish the mechanism of action (MoA) of novel screening hit.

## Introduction

The human pathogen, *Mycobacterium tuberculosis* (*Mtb*) is the causative agent of tuberculosis (TB), an infectious disease that is widespread, infecting around one third of the world’s population [1]. The discovery of streptomycin in 1943, and the subsequent discovery and optimization of other anti-tubercular drugs, such as *para*-aminosalicylic acid, pyrazinamide, cycloserine and ethambutol, and the introduction of directly observed short-course chemotherapy (DOTs) delivered initial significant patient and societal benefit. However, the recent emergence of multi-drug resistant (MDR) and extensively drug-resistant (XDR) strains of *Mtb* [2], as well as co-infection with HIV, and extended duration of chemotherapy and diagnostic delays [3], have led to the re-emergence of TB as a global health threat. The worldwide mortality rate of TB is more than 1.4 million people per year, and it is the second leading cause of death from a single infectious agent after HIV [1]. In 2012, around 13% of the 8.6 million people who had developed TB were HIV-positive, and 75% of these cases were in Africa [4].

To date, a variety of methods are currently employed to identify new drug leads differentiated from previous therapies, in addition to targeting an essential process in the bacteria, such compounds also need to overcome several specific problems associated with TB drug development, such as the significant permeability barrier, combat MDR and XDR TB, and underlying safety profiles when used in conjunction with other drugs, in the case of co-infection with HIV. Additionally, commercial and regulatory aspects have not provided sufficient investor-led interest in development of novel *Mtb* drugs. This has however led to a combined effort from worldwide academia and industry on several collaborative partnerships to find solutions to this developing TB crisis.

High-throughput screening (HTS) is one method being used to identify new drugs from large compound repositories [5]. In this regard, GlaxoSmithKline (GSK), has identified and released the activities and structures of a large set of anti-mycobacterials into the public domain; these are available in the ChEMBL database [6] (https://www.ebi.ac.uk/chembl/). This dataset consists of 776 anti-mycobacterial phenotypic hits with activity against *M*. *bovis* BCG. Amongst these, 177 compounds were confirmed to be active against *Mtb* H37Rv (MIC < 10 μM) and also displayed low human cell-line toxicity [7]. These whole-cell hits provided a privileged set of compounds with the ability to cross the cell wall of *Mtb*, overcoming one of the major challenges for orally administered TB drugs [8–10]. However, the mode of action (MoA) of these compounds is yet to be elucidated. The identification and validation of the molecular target(s) of a compound is a complex and yet fundamental strategy in the drug discovery [11]. Consequently, it is important to develop novel, and improve on existing, methods currently used to identify and validate targets for bioactive compounds.

Advances in integrative computational methodologies combined with chemical and genomics data offers a multifaceted *in silico* strategy for efficient selection and prioritization of potential new lead candidates in anti-TB drug discovery. Utilising chemical, biological and genomic databases enables the development and usage of computational ligand-based and structure-based tools in the discovery of TB targets linked to the MoA studies. Recently, chemogenomics, an approach that utilizes chemical space (physical and chemical properties) of small molecules and the genomic space defined by their targeted proteins to identify ligands for all targets and *vice versa* [12], Structure Space and Historical Assay Space approaches have been used to determine the MoAs for the aforementioned published GSK phenotypic hits [13]. This initiative has paved the way to an array of computational target prediction approaches for TB. To date, 139 compounds were predicted to target proteins belonging to diverse biochemical pathways. In addition, *TB mobile*, [14] platforms has been used to predict targets for these phenotypic hits. Targets predicted from both methods include essential protein kinases and proteins in the folate pathway, as well as ABC transporters. Although, these methods provide valuable information on potential targets of anti-TB compounds identified in phenotypic screens, no *in vitro* validation of the *in silico* modeled targets has been so far reported.

We have applied two distinct ligand-based computational approaches in conjunction with a structure-based approach (docking) to predict potential targets for an anti-TB phenotypic hit series. To increase likely prediction accuracy we applied a tournament of three distinct methods, which we believe complement each other. For the first time, we present the *in vitro* validation of these results for the predicted target-compound interactions involving the *Mtb* dihydrofolate reductase (DHFR). *Mtb* DHFR is an essential protein that catalyses the reduction of dihydrofolate to tetrahydrofolate (THF), a co-factor in the production of thymidylate, purine bases and amino acids important for the synthesis of DNA, RNA and proteins [15,16]. There are no drugs presently in clinical use that target this enzyme for *Mtb*, therefore this work provides experimentally confirmed ligands for mycobacterial DHFR, which will serve as starting points for further hit-to-lead optimisation. In addition, our studies present computational and experimental approaches that can effectively characterize and prioritize phenotypic assay hits.

## Materials and Methods

### Ethics Statement

All experiments were approved by EMBL-EBI, University of Birmingham, and Diseases of the Developing World (DDW-GSK) ethical committee where required and there are no ethical issues to report.

### Compound preparation

A set of the 776 TCAMS-TB dataset [7] compounds was retrieved from the ChEMBL database (https://www.ebi.ac.uk/chembl/). Using suitable protocols in Pipeline Pilot version 8.5 from Accelrys [17], 2D coordinates for each compound were generated from their canonical SMILES. The stereochemistry, charges (formal charges for common functional groups e.g. quaternary nitrogen, nitro groups, *etc*. were standardized, salts and single atom fragments were removed, and the largest molecular fragment was kept.

### Target Prediction using Multiple Category Naive Bayesian Classifier

#### Model Generation and validation

ChEMBL is a database of bioactive small molecules that contains 2D structures, calculated physical chemical properties and abstracted bioactivity data extracted from scientific journals [18]. ChEMBL version 17 (http://www.ebi.ac.uk/ChEMBL), contains more than 1.3 million compound entities, > 9,000 annotated targets, > 50,000 publications and more than 11.4 million reported activities. The database was queried to collate a dataset of 698,401 human and bacterial target-ligand pairs, (covering 2,257 unique proteins in total) with the standard activity types and values defined as IC_50_, EC_50_ or K_i_ ≤ 10 μM, or inhibition ≥ 50% together with information of their experimental documentation. Retrieved targets, identified by their Uniprot accession numbers, were proteins with target confidence scores equal to or greater than 7. This is a score in ChEMBL that shows the level of confidence in assigning molecular target, where scores 7, 8 and 9 indicate direct assignment to protein complexes, homologous single protein and single protein, respectively. From the retrieved SMILES of the ligands, molecular structures were generated and standardized using a Pipeline Pilot protocol.

To increase the strength of the multiple category naïve Bayesian classifier models (MCNBCs), the dataset was filtered and 695,902 target-ligand pairs containing 1,543 targets assigned to at least 10 ligands were collected. For each protein accession number, the MCNBCs were trained on the structural features of all compounds using a Pipeline Pilot protocol, in conjunction with the extended-connectivity fingerprints of diameter 6 (ECFP_6) [19]. These circular fingerprints are intended to identify precise atom environment sub-structural features, limited to a maximum radius of 3 bond lengths, in a molecule and have been successfully utilized in similarity ligand–based virtual screening of small molecule databases [20] and in TB target prediction [**13**],[21].

The efficiency of the model was determined by firstly, training a model on randomly selected 80% of the compounds consisting of 1,543 proteins associated with 556,188 compounds, and EFCP_6 fingerprints. The model was tested using 52,809 unique compounds from the remaining 20% of the dataset. This approach guaranteed the randomized selection of compounds for both the training and test sets and minimized bias by presenting the model with a test set of previously unseen compounds. Here the different categories/proteins are learned by considering the frequency of appearance of a particular sub-structural feature for their different ligands [13,22,23]. The naïve Bayesian (NB) score is based on the Bayes rule of conditional probability which states that for two given events A and B the probability of A occurring, given that B has already occurred, P(A|B) is given by P(A|B) = P(B|A) P(A)/P(B) where P(A) and P(B) are probabilities of A and B respectively [22]. The probabilities are calculated using the Laplacian-corrected estimator. More specifically, the NB score of a target is the sum (P_total_) of the logarithm of Laplacian-corrected Bayes rule of conditional probability [P(A|F_i_)] for each fingerprint feature of a compound [13]. The predicted targets are ranked based on their NB scores, in descending order. The efficiency of the model was indicated by the calculated percentage of compounds with correctly assigned targets reported in ranked positions 1–5.

To avoid bias through inclusion of closely related compounds to the training set, compounds from randomly selected 80% articles (in which the compounds and bioactivity data were published), were used to train a second model. This training set consisted of 1,505 proteins associated to 586,928 diverse compounds. The model was tested using unique compounds retrieved from the remaining 20% of the articles, and the set contained least 108,974 molecules. This approach guaranteed selection of random and diverse compounds for both the training and test sets. For each target, the total Laplacian-corrected normalised probability [13,23] for all compound features was calculated and reported as the NB score. The predicted targets were ranked based on their NB scores, in descending order. In both cases the efficiency of each model was determined by calculating the percentage of compounds with correctly assigned targets reported in positions 1–5. In addition, the models were validated using “leave-one-out” cross-validation, in which each sample was left out and a model built using the rest of the samples. The model was then used to predict targets for the left out sample.

### MCNBC Target Prediction

To predict targets for the 776 anti-mycobacterial compounds, a model trained on all 695,902 target-ligand pairs from ChEMBL was built. Targets with less than 10 annotated ligands were excluded from the training set. Predicting targets for > 1,200,000 compounds in ChEMBL version 17 database generated the background information by calculating mean NB scores (μ) and standard deviation (σ) for each target. The entire 776 compounds were tested against the 100% model and NB scores for each target were calculated for each compound. For each predicted target, standard scores (Z-score) were calculated from a statistical analysis of the NB scores for each compound; Z-score = (X—μ)/σ where, X is the NB score of a target for a compound. The Z-score distinguishes the compound scores for a particular target from the influence of the background noise. The predicted targets were then filtered and compounds with positive NB scores and Z-scores > = 1.5 were retained. Suggested targets for each compound were then ranked using NB scores.

### Using Similarity Ensemble Approach (SEA)

SEA utilizes chemical similarity between two sets of ligands to study the pharmacological relationships between drugs. In previous work, the tool was used to predict new on- and off- targets and adverse drug reaction for known drugs that were later confirmed experimentally [24],[25]. We have used the SEA search tool available at sea.bkslab.org, to predict targets for all 776 GSK phenotypic hits. To validate the method, the tool was used to predict targets for TB drugs of known modes of action but for which the bioactivity data is not found in ChEMBL version 16. The anti-TB dataset was divided into smaller input files of approximately 100 compounds represented by their SMILES strings and identifiers. Similarity search between ligand sets was derived from ChEMBL version 16 based on EFCP_4 fingerprints. In this method the aggregate Tanimoto similarity score, is converted, using a statistical model, to the expectation (*E*) value that describes the significance of similarity between an orphan compound and a set of ligands and hence its most likely targets [26]. In addition to predicting multiple targets from different organisms for a given compound, SEA also predicts targets at different ligand activity levels (10,000; 1,000; 100; 10 and 1 nM). For our purposes, the best (smallest) *E*-value for each target- compound pair was acquired and predictions with expectation values less than 10^–1^ were significant.

### Using Structure-based approach (docking)

The ligand-based methods were here used o streamline the *Mtb* proteins for docking calculations. The Internal Coordinate Mechanism (ICM) method developed by Molsoft L.L.C [27] was used to generate binding modes of the small molecules in the binding pocket of selected proteins and to estimate the strength of the protein-ligand interactions based on the ICM scoring function: ΔG = Δ*E*_IntFF_ + *T*Δ*S*_Tor_ + α_1_Δ*E*_HBond_ + α_2_Δ*E*_HBDesol_ + α_3_Δ*E*_SolEl_ + α_4_Δ*E*_HPhob_ + α_5_ *Q*_Size_ where: Δ*E*_IntFF_ is change in van der Waals interactions of ligand and receptor and the internal force-field energy of the ligand, *T*Δ*S*_Tor_ is the change in free energy due to conformational entropy and weighted (α_1_ – α_5_), Δ*E*_HBond_ is the hydrogen bond term, Δ*E*_HBDesol_ accounts for the disruption of hydrogen bonds with solvent, Δ*E*_SolEl_ is the solvation electrostatic energy change upon binding, Δ*E*_HPhob_ is the hydrophobic free energy gain and *Q*_size_ is the ligand size correction term. The ICM scores were standardized by determining the ligand efficiency indices (LEI) (ICM score divided by the number of heavy atoms) for each docked molecule [28].

Dihydrofolate reductase (DHFR), an enzyme important in the last stage of tetrahydrofolate biosynthesis, was identified as a potential target for the 776 phenotypic hits. The protein has been widely studied and there are three crystal structures of the open conformation of the enzyme in complex [16] with cycloguanil (PDBe 4kne, 2.00Å resolution), trimethoprim (PDBe 4km2, 1.40Å resolution) and pyrimethamine (PDBe 4km0, 1.30Å resolution) in the Protein Database in Europe (PDBe). Based on the percentile ranks reported in PDBe (http://www.ebi.ac.uk/pdbe-srv/view/entry), these three have better crystal structure quality compared to other structures. The coordinate files for 4kne, 4km0 and 4km2 were retrieved and the ligand coordinates were saved in separate files. Using ICM-docking receptor preparation tools the three protein structures were separately prepared by deleting all water molecules, optimize hydrogen, adding missing heavy atoms and hydrogen, and adjusting amide groups and were saved as ICM receptor molecules. The “setup receptor” tool was used to generate receptor maps using a grid size of 0.5Å. Similarly; the cycloguanil, trimethoprim and pyrimethamine structure files were prepared and converted to ICM molecules. To validate the docking calculations the three prepared ligands were re-docked into their respective protein structures, using default ICM parameters and a thoroughness/an effort value of 2 regulated the length of the docking simulation [29]. The generated binding conformations were compared to the crystal structure conformations. Cycloguanil gave the best conformation, RMSD = 0.3 Å (ICM score = -25.79) (S1 Fig., supporting information) compared to pyrimethamine (RMSD = 1.46 Å) and trimethoprime (RMSD = 0.86 Å). The crystal structure of DHFR in complex with cycloguanil (4kne) was therefore used in the production stage.

Three dimension coordinates of 776 anti-TB compounds were generated using a Pipeline Pilot protocol and saved as mol2 files. The molecules were imported into ICM, amide bonds were fixed, hydrogen atoms were built and the structures were converted into ICM molecules. The compounds were docked into 4kne using thoroughness/effort value of 2 and default ICM parameters. The compounds were ranked on their LEI and the top 100 compounds were retained for further analysis. Compounds with MIC (BCG) < 5.00 μM that were commonly predicted to inhibit DHFR or had LEI >1.00 were selected for in vitro validation.

### Generation of *M*. *bovis* BCG resistant mutants, WGS, over-expression and MIC determination

Chemical compounds used in this work were supplied by GSK. The generation of spontaneous resistant mutants and WGS was conducted as described in [30]. For the over-production of ThyA and DHFR in *M*. *bovis* BCG, the corresponding genes were cloned into pMV261. The genes were initially amplified by PCR (Phusion High-Fidelity DNA polymerase; New England Biolabs) from *M*. *bovis* BCG strain Pasteur genomic DNA. The oligonucleotide primers used are shown in Table 1. The fragment sizes 0.8 kb (*thyA*) and 0.5 kb (*dfrA*) were cloned into the pMV261 vector by exploiting the primer encoded restriction sites *Bam*HI and *Hind*III (FastDigest restriction endonucleases, Fermentas; T4 DNA ligase, New England Biolabs). The constructs were verified by DNA sequencing. The constructs, including the empty pMV261 vector were electroporated into *M*. *bovis* BCG and MIC values determined as described [30].

**Table 1:** Primers used in the generation of constructs pMV261::*dfrA* and pMV261::*thyA*.

Primer	Sequence (5’-3’)
*dfrA* sense	CATGCATGGATCCGATGGTGGGGCTGATCTGG
*dfrA* anti-sense	CATGCATGAAGCTTAATCATGAGCGGTGGTAGCT
*thyA* sense	GATCGATCGGATCCAGTGACGCCATACGAGGACCTGCTG
*thyA* anti-sense	GATCGATCAAGCTTTCATACCGCGACTGGAGCTTTGATCGC

Restriction sites used in the cloning procedure are underlined (*Bam*HI, *Hind*III)

## Results

### GSK phenotypic hits as ligands

The 776 GSK screening file phenotypic hits, available from the ChEMBL database (https://www.ebi.ac.uk/chembl/) were used as input structures for target prediction using three distinct computational approaches. *Mtb* targets for this set of compounds have been previously proposed, where three different laboratories combined structural, historical and chemogenomic data to predict their MoA [**13**]. A broad spectrum of predicted targets was proposed but no experimental validation was reported. A detailed account of the physicochemical properties and similarity clusters of the 776 compounds has been reported previously and is not covered in this work. However, it should be noted that more than 90% of the compounds fall within the acceptable range for drug-like compounds [13].

### Ligand and structure-based target prediction approaches

Two ligand-based methods were used to enable target prediction based solely on ligand 2D properties in the absence of target structural information. Multiple category naïve Bayesian classifiers (MCNBC) have been extensively applied in target prediction studies [13], [22], [21]. A second distinct method—Similarity Ensemble Approach (SEA) is widely used to predict targets based on similarities between a compound of unknown MoA and ligands sets with known targets [26]. To complement the two ligand-based methods, we also used a structure-based approach that enables the use of available structural information of a known target to identify compounds whose 2D molecular features are absent in known ligands and are low ranked compounds by MCNBC and SEA. Hence, selected targets identified from MCNBC and SEA were used in a structure-based strategy involving docking calculations of candidate compounds, in order to investigate their binding as defined by the binding site occupancy, orientation, non-covalent bond interactions and their ligand efficiency index (LEI).

### Exploring genomic space based on 2-D chemical space of ligands

**Fig 1:**
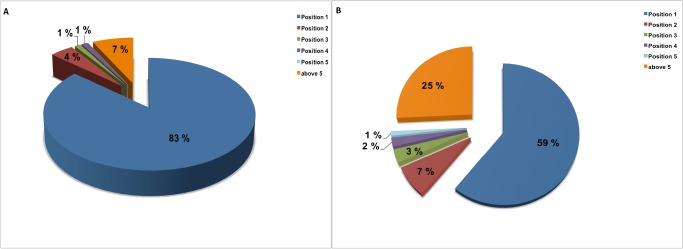
The multiple category naïve Bayesian classifier validation results. (A) Validation results of the model generated from randomly selected 80% target-ligand pairs and the remaining 20% was used as a test set. (B) Validation results of the model built using target-ligand pairs from 80% of the published articles and the test set consisted of target-ligand pairs from the remaining 20%.

A prediction algorithm was created that employed a multiple-category naïve Bayesian classifier (MCNBC) model and the 2D ECFP_6 fingerprints [31] of compounds in the ChEMBL database with pre-established inhibitory activity. Upon validation, the generated MCNBC was able to correctly assign targets to ~93% of the compounds (Fig. 1A). These compounds had their annotated targets assigned to rank positions 1–5 indicating high model accuracy. It is important to note that a similar target prediction strategy was recently utilized to help suggest target-ligand pairs for the same set of compounds active against *Mtb* and it had over 90% prediction accuracy (as judged by correct target identified in the top scoring 5 predictions) in training [13]. The main difference between this method and the version presented in this study is that our training data consisted of human and biological target-ligand pairs; furthermore, in order to increase the target coverage of the models, each target had at least 10 ligands, instead of the previously reported 40 or more ligands for a given target [13]. Even though we used targets with as few as 10 reported ligands, comparable validation results were obtained. The second validation procedure, reported here for the first time, involved randomly splitting about 15,720 documents into 80% and 20% sets and using target-ligand pairs in the 80% document set to train a second model—typically the boot-strapping approaches previously used do not split by chemical series (approximated here by the reporting of congeneric series in an individual publication), we therefore consider our validation approach as more indicative of real-world applications. This way a selection of random and diverse compounds for both the training and test sets was guaranteed. Considering the high diversity of the chemotypes in our test set, the model achieved a satisfactory recall of 75% (Fig. 1B) upon validation. In addition to the aforementioned validation approaches, the “leave-one-out” cross-validation, in which each sample was left out and a model built using the rest of the samples was used. For all the categories in the model, the calculated receiver-operator characteristic (ROC) area under the curve (AUC) [9] score was greater than 0.7 and most models retrieved at least 80% of their ligands within 10% retrieval of the dataset, indicating high sensitivity and specificity in assigning the correct categories/targets. Subsequently, a final MCNBC model was generated using 100% of the human/bacterial target-ligand pairs from ChEMBL version 17.

Ligand–based approach can involve activity profile similarity or comparison of chemical similarity between a compound and a set of reference ligands [32]. SEA utilizes chemical structural similarity between two sets of ligands to infer protein similarity. The output is an expectation (*E*) value statistically derived from the sum of the Tanimoto similarity of the substructural fingerprints of all pairs between the anti-TB compounds and sets of ligand for given targets. A smaller statistically derived *E* value indicates a stronger similarity between two proteins and hence potential targets.

Flouroquinolones, antibacterials known to inhibit DNA gyrase and topoisomerase IV [33,34] whose target-ligand pairs were *not* in ChEMBL version 17 were presented to the MCNBC model and SEA for further validation. The two ligand-based methods correctly assigned gatifloxacin, ofloxacin, moxifloxacin and lexofloxacin to *Staphylococcus aureus* topoisomerase IV (UniProt accession: P0C1U9). From the top five predictions using SEA, topoisomerase IV was found in position one and *E*-values ranged from 2.20E-46 for moxifloxacin to 2.05E-27 for lexofloxacin and ofloxacin. Using the MCNBC model, the correct known target was in positions 1 and 2 for gatifloxacin (Z-score = 6.35) and moxifloxacin (Z-score = 7.99) respectively, and in eighth position for ofloxacin and lexofloxacin both displaying a Z-score of 3.63. Based on these observations, MCNBC model and SEA were therefore used to predict targets for the 776 novel anti-tubercular compounds.

### Number of predicted targets

Both MCNBC and SEA are tools that can be used to propose an ensemble or set of likely biological targets for new bioactive compounds and the results can indicate potential on-target polypharmacology and off-target side effects of the drugs as well as phenotypic hits. Based on the 2D chemical space, defined by ECFP_6 fingerprints [31] of each of the 776 GSK hits, MCNBC predicted 1,462 targets, all with positive Bayesian scores (NB) and Z-scores > = 1.5, possibly defining the bioactivity space of the compounds. The most frequent targets were for the *Homo sapiens* proteins, which constituted about 90% (1313 proteins) of the predicted targets whilst bacterial proteins made up approximately 10% (146 proteins). There were a total of 25 unique proteins in our training set spanning from kinases, (e.g serine/threonine-protein kinases like PknB, reductases like enoyl [acyl-carrier-protein] reductase (InhA), transcriptional regulators (HTH-transcriptional regulator EthR) hydrolases (like Epoxide hydrolase, EphB), that were assigned 132 compounds (S2 Fig., supporting information). *Mtb* drug targets were further inferred by mapping functional data and chemical bioactivity data of all predicted targets across their *Mtb* orthologues based on the OrthoMCL database [35]. This approach has been used elsewhere to identify potential pathogenic drug targets [**13**,^36^]. The final number of identified *Mtb* targets was 119 for 698 compounds (2343 target-ligand pairs) (Data is available on request). For each compound, the predicted targets were ranked according to their Z-scores. About 23 compounds were predicted as modulators of *Mtb* DHFR (UniProt accession: P9WNX1 (previously P0A546)).

SEA assigned 36,607 target-ligand pairs for 1346 proteins, from all the organisms in ChEMBL version 16. Most compounds were assigned to human targets (~79% of predicted targets) and 13 *Mtb* proteins were predicted. This number was increased to 110 after considering the *Mtb* orthologues resulting in 1333 target-ligand pairs (428 compounds). In agreement with MCNBC predictions, *Mtb* DHFR was one of the proteins identified to be a potential target for 17 phenotypic hits. The two methods commonly predicted 12 *Mtb* DHFR inhibitors.

### Supporting 2-D based predictions with docking

A structure-based approach was used to identify potential ligands based on the normalized binding score and determine the binding modes of the phenotypic hits to *Mtb* DHFR. The entire 776 anti-TB compounds were docked into the binding pocket of DHFR using the Internal Coordinate Mechanism (ICM) method [27] and the strength of binding interactions decreased from compound GSK1839228A (ICM score = -35.00 to GSK1452001A with ICM score of-4.20. Eighteen compounds displayed ICM scores higher than that of cycloguanil (ICM score > 25.00) indicating stronger binding interactions. The docked phenotypic hits were listed in descending order of their Ligand Efficiency Index (LEI), calculated by dividing the ICM score for each compound by the number of heavy atoms. The top 100 compounds, whose LEI ranged from 0.78 to 1.45, (S1 Table, supporting information) were retrieved as the high ranked docking compounds (hits) and were used in further analysis.

### Predicted *Mtb* DHFR ligands

**Fig 2:**
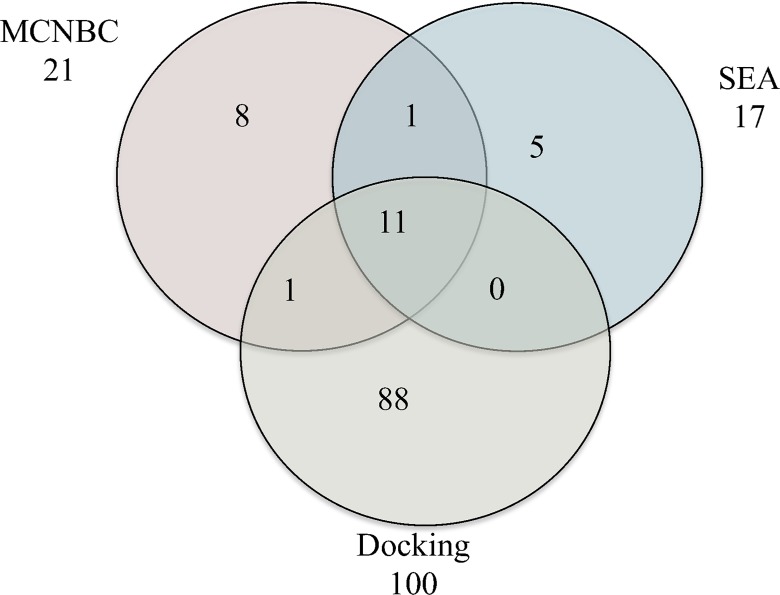
Number of potential inhibitors of *Mtb* DHFR identified by the multiple category naïve Bayesian classifier (MCNBC), Similarity Ensemble Approach (SEA) and docking calculations.

The total number of *Mtb* DHFR ligands according to predictions by MCNBN and SEA was 28 (S2 Table, supporting information) and 12 of these compounds were amongst the top 100 molecules proposed by our structure-based approach (Fig. 2). Although both SEA and docking approaches recognized no common modulators, it was encouraging that the three orthogonal methods commonly identified eleven potential inhibitors for *Mtb* DHFR (Fig. 2, S2 Table, supporting information). Out of these, eight compounds, **S4**, **S5**, **S6**, **S8**, **S11**, **S12**, **S20**, and **S21** (S2 Table, supporting information) contain the 1,4,5,6-tetrahydro-1,3,5-triazin-2-amine (THT) and the phenoxy-propoxyl scaffolds, present in the potent *P*. *falciparum* DHFR inhibitors, cycloguanil and WR99210 [16,37]. The two scaffolds occupied important binding positions in DHFR binding pocket, and formed hydrophobic and hydrophilic interactions with the residues. For instance, the THT moiety is known to interact with Asp27, a residue important for activation of DHFR [37]. Molecular structures of compounds **S1**, **S25** and **S22** consist of novel DHFR inhibitor scaffolds, which are the methoxyisoquinolin-8-yl, the substituted quinoloin-5-amine and the quinazoline-2,4, diamine respectively. The overlap between MCNBC and SEA consisted of compound GW351921X (Z-score = 3.14 and E-value = 2.38E-14). This compound had a LEI of 0.74 and did not appear in the top 100 set from docking calculations since the LEIs are lower than 0.78. The single overlap between MCNBC and docking predictions is a small compound **S10** (GSK747165A) made up of the methoxyphenyl substructure linked to a phenyl ring. About 38% (8/21) of the MCNBC predicted ligands were solely suggested, SEA had 29% (5/17) and out of the 100 docking hits, 88% (88/100) were exclusively proposed (Fig. 2).

The structure-based approach also identified potential ligands containing chemical entities not commonly found among DHFR inhibitors, such as methotrexate **(S29)**, trimethoprime **(S30)**, cycloguanil **(S31)** and WR99210 **(S32)** [37] (S3 Fig., supporting Information). 3-Chloro-(methoxyphenyl)benzene-1, 2-diamine, (LEI = 1.45), is a small molecular weight (248.7) compound with two substituted aromatic rings and 2-(2-phenoxyethoxy)-5-(thiophen-3-yl)-benzamide, also displays novel chemical features and has a molecular weight of 339.41. The third compound, GSK747165A (**S10**) (LEI = 1.41, Mwt = 214.26), 1-N-(4-methoxyphenyl)benzene-1,2-diamine, is an analogue of 3-chloro-(methoxyphenyl)benzene-1, 2-diamine that lacks the chloro-substituent. All highly ranked potential *Mtb* DHFR inhibitors derived by docking but compound 2-(2-phenoxyethoxy)-5-(thiophen-3-yl)-benzamide were tested in *in vitro* experiments against *M*. *bovis* BCG.

### BRL-7940SA and BRL-10143SA inhibit mycobacterial DHFR

In recent years, whole genome sequencing (WGS) of spontaneous resistant mutants has been a successful tool in identifying the target of various anti-tubercular compounds [38]. This tool was used to test potential DHFR inhibitors that initially displayed MIC (BCG) < 5 μM, such as BRL-7940SA (**S5),** BRL-10143SA (**S12),** GW369335X (**S22)**, and GSK747165A (**S10**) (S2 Table, supporting information) against *M*. *bovis* BCG. Two tetrahydro-1,3,5-triazin-2-amine (THT) derivatives, BRL-7940SA, 5-(3-(2-(*tert*-butyl)phenoxy)propoxy)-4-imino-6,6-dimethyl-1,4,5,6-tetrahydro-1,3,5-triazin-2-amine (THT1) (**S5**), and BRL-10143SA 5-(3-(2-ethylphenoxy)propoxy)-4-imino-6,6-dimethyl-1,4,5,6-tetrahydro-1,3,5-triazin-2-amine (THT2), (**S12**), displayed inhibitory activity against *M*. *bovis* BCG DHFR.

**Fig 3:**
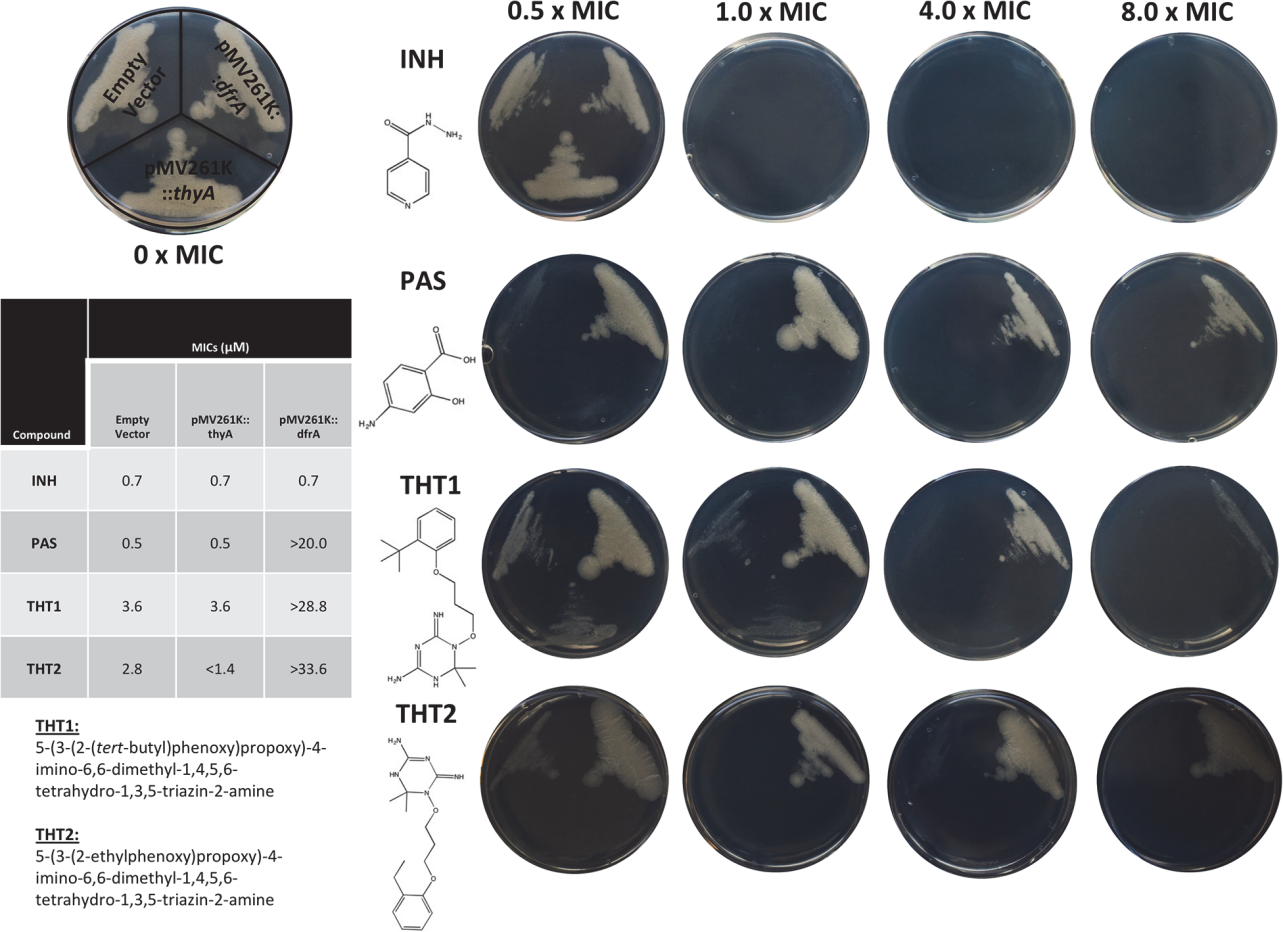
Impact on the MIC of THT1 and THT2 upon the over-expression of ThyA and DHFR with Isoniazid (INH) and *para*-aminosalicylic acid (PAS) as negative and positive controls. The MICs of THT1 and THT2 were determined in *M*. *bovis* BCG containing pMV261, pMV261::*thyA* and pMV261::*dfrA*. Plates are shown at 0.5, 1.0, 4.0 and 8.0 x MIC of each compound (with respect to the empty vector), the structures of which are given along with the IUPAC nomenclature for THT1 and THT2 and tabulated MIC data.

The MIC of THT1 was established in *M*. *bovis* BCG to be 3.6 μM. Spontaneous resistant mutants of *M*. *bovis* BCG were generated at 5 x MIC with a mutational frequency of 4 x 10^8^. WGS of one of these spontaneous resistant mutants revealed two high quality single nucleotide polymorphisms compared to the sequenced wild-type *M*. *bovis* BCG strain and reference sequence. Both mutations, in *thyA* (W80S) and PPE5 (G476D) were detected with 100% allele frequency. Mutations in ThyA (thymidylate synthase) have been linked to resistance against the confirmed DHFR inhibitor, para-aminosalicylic acid (PAS) [39]. Consequently, over-expression studies of ThyA and DHFR in *M*. *bovis* BCG were performed to confirm the target of THT1 and to determine the impact on the MIC of the remaining *in silico* identified compounds (Fig. 3). There was no increase in resistance upon over-expression of DHFR or ThyA on the negative control, isoniazid (target: InhA) (Fig. 3). Only the DHFR over-expresser strain exhibited an increase in resistance when tested on the positive control, PAS as shown by the MICs given in Fig. 3 (target: DHFR). Over-expression of DHFR on THT1 and THT2 resulted in an increase in MIC from 3.6 μM to >28.8 μM and 2.8 μM to >33.6 μM, respectively. Conversely, the ThyA over-expresser strain did not give any resistance to THT1 (MIC 3.6 μM) for both ThyA over-expresser and empty vector, and gave increased sensitivity to THT2 from 2.8 μM to <1.4 μM.

## Discussion and Conclusion

Chemogenomics approaches have provided fast and cheap utilization of the chemical and genomic space in identification of target-ligand pairs that were confirmed by using WGS methods, followed by over-expression of ThyA and DHFR in *M*. *bovis* BCG. To our knowledge, this is the first time computationally predicted mycobacterial target-ligand pairs have been phenotypically validated. Compounds **S4** and THT2 (**S12)** have been reported to potentially modulate the folate pathway [**13**]. Here, compounds THT1 (**S5**) and THT2 (**S12**) have been confirmed to target mycobacterial DHFR. Three distinct, yet complementary, *in silico* methods independently predicted the two compounds.

**Fig 4:**
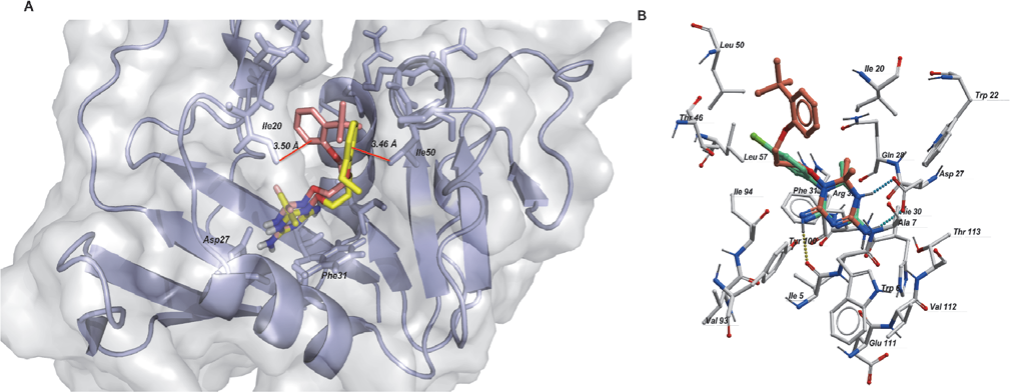
Binding mode of BRL-7980SA (THT1) and BRL-10143SA (THT2). (A) Grey defines the molecular surface and the cartoon represents the secondary structure of *Mtb* DHFR. Sticks represent bonds and atoms of THT1 (brown), THT2 (yellow) and the binding pocket residues within a radius of 5 Å from the ligand. (B) A comparison of binding mode of THT1 and cycloguanil (green).

**Fig 5:**
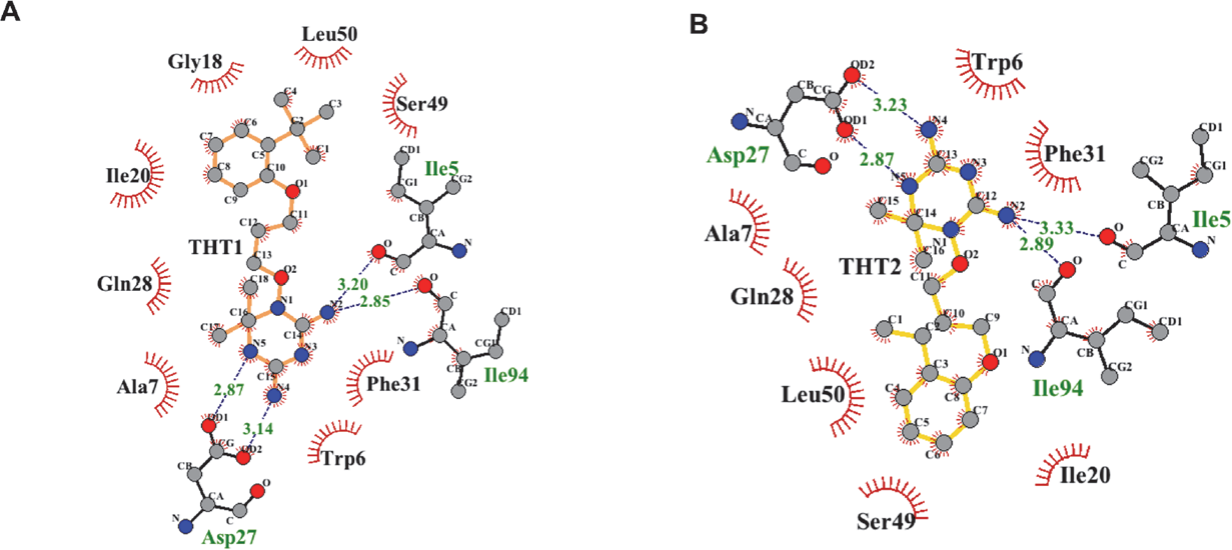
Interactions of (A) THT1 and (B) THT2 with binding pocket residues in DHFR. Residues in close contact and interacting through hydrophobic interactions are shown in red and H-bonds (green dots) and their distances are in green. Black lines depict residues forming polar contacts with the ligand.

In docking calculations involving *Mtb* DHFR, the two compounds have similar orientation in the binding pocket (Fig. 4A), similar to the binding modes of cycloguanil (Fig. 4B), methotrexate, trimethoprim and Br-WR99210 previously reported [37]. The THT moiety in THT1 (brown sticks) and THT2 (yellow sticks) occupied the inner hydrophobic binding site bordered by, amongst other residues, Phe31 and forms H-bonds with Ile5 and Asp27 and Ile94 as well as hydrophobic interactions (Fig. 5). The *ortho*-substituted phenyl ring occupies the outer hydrophobic binding site close to the entrance of the pocket and form van der Waals forces (London forces) with these residues with residues Gly18, Ile20, Thr46, Ser49, and Leu50 (Fig. 5). In this site there are differences in orientation where the phenyl ring in THT1 is drawn closer to Il320 and closest distance between them is ~3.50 Å, whilst the *tert*-butyl fragment interacts more with Leu50 (Fig. 5A). In contrast, the ethyl-phenyl- moiety of THT2 is closer to Leu50 (closest distance ~3.49 Å) and there is minimum contact with Ile20 (Fig. 5B). Largely, both molecules are stabilized by hydrophobic and polar interactions.

DHFR is essential for the production of tetrahydrofolate [16] that is crucial for the synthesis of DNA and proteins. Inhibition of this enzyme could lead to cell death and therefore inhibit the growth of *Mtb*. It is important to note that THT2 was also predicted to target InhA, Phenylalanine–tRNA ligase alpha subunit, and Fibronectin-binding protein C. On the other hand THT1 was also predicted to target dihyropteroate synthase 1 (DHPS) and Phenylalanine—tRNA ligase alpha subunit. In our predictions the *Mtb* DHFR was inferred from its orthologous genes that included DHFR from *Homo sapiens*, *Bacillus anthracis*, *Escherichia coli*, *Lactococcus lactis*, *Staphylococcus aureus*, *Neisseria gonorrhoeae and Lactobacillus casei*. Therefore, orthology proved to be a significant tool that can be used to link a known drug target with a potential novel target [36].

Clearly, following chemogenomic approaches to predict a given compound’s molecular targets has the potential to reveal alternative ligands for existing targets for *M*. *tuberculosis* infection and other diseases. Such approaches can also suggest new targets for new drugs and de-convolute their adverse drug reactions (ADR). Nevertheless, the limitations of using such models based on the ChEMBL database include the fact that, in general, the predictions do not distinguish between agonists/activators or antagonists/inhibitors; however, the activities of the compounds have been confirmed through experimental validation. In the absence of such corroboration, activities can be inferred based on the predicted targets and compound structure. Additionally, since both the MCNBC and SEA target prediction models are trained on the ChEMBL database of known target-ligand pairs, all predicted targets are biased towards previously studied and reported proteins. Thus, this method is not able to predict directly new, unprecedented, protein targets in biological pathways that haven’t been thoroughly studied and added to the ChEMBL database. This limitation is however overcome by the recurrence of already validated targets, but with distinct and novel chemotypes from phenotypic screening studies coupled with the consideration of predicted *Mtb* protein orthologues and *in vitro* validation.

**Fig 6:**
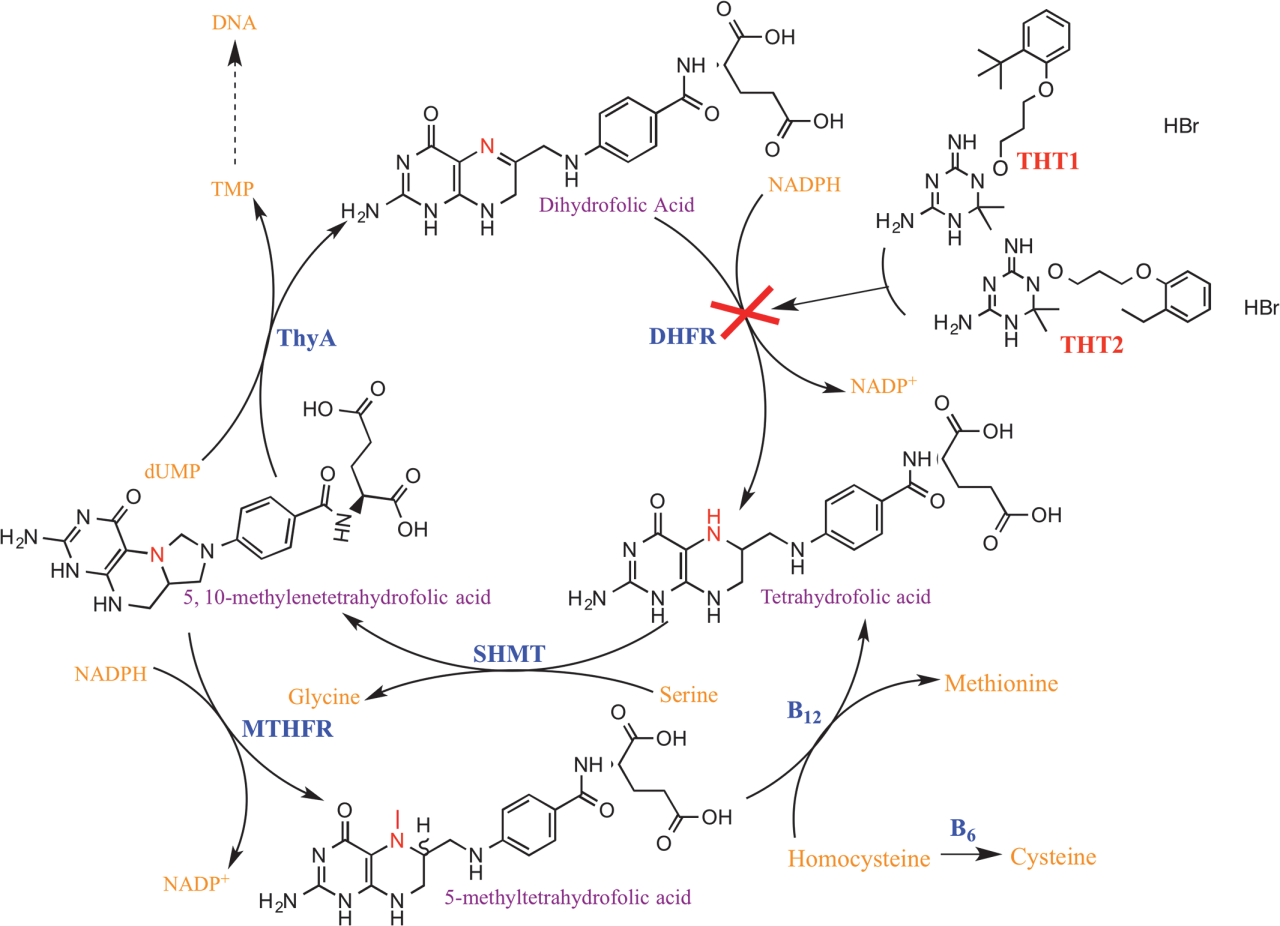
The biochemical relationship between ThyA and DHFR. Enzymes are highlighted in blue. ThyA, thymidylate synthase; DHFR, dihydrofolate reductase; SHMT, serine hydroxymethyltransferase; DHF, dihydrofolate; THF, tetrahydrofolate; mTHF, methyl tetrahydrofolate.

The over-expression studies confirm DHFR as the target of THT1 and THT2: increased target levels enable the cells to survive in higher concentrations of drug. In the folate biosynthetic pathway, DHFR generates tetrahydrofolates from DHF, the derivatives of which are consumed by ThyA with the conversion of dUMP to dTMP (for DNA synthesis) and the regeneration of DHF. This cycle ensures the replenishment of the intracellular stores of THF derivatives, which are used in other essential single-carbon transfers. Inhibition of DHFR results in a reduced production of THF, which is readily used by a major consumer of reduced folates, ThyA, causing a depletion of the stores of THF (Fig. 6). Over-expression of ThyA on the DHFR-targeting compounds THT2 resulted in an increased sensitivity to the compound. The increased cellular levels of ThyA would cause a greater turnover of THF, the replenishment of which would be further limited by the inhibition of native levels of DHFR in the cell by the compound. The mutation in ThyA in the spontaneous resistant mutant locates to the active site. It is probable that the mutation causes a reduction in thymidylate synthase activity. Therefore, more folates are available for essential one-carbon additions. ThyX is a functional analogue of ThyA [39]. ThyX bypasses the ThyA/DHFR pathway and is involved in *de novo* thymidylate synthesis [40] compensating for the reduced activity of ThyA.

Therefore three different but complimentary computational methods were used to identify inhibitors of *M*. *bovis* DHFR. Two out of eight compounds (giving a hit rate of 25%) were confirmed using *in vitro* Whole Genome Sequencing experiments. This work provides two inhibitors, THT1 and THT2 that can be modified into selective *mycobacterium* DHFR inhibitors or used as chemical probes in biological systems.

## Supporting Information

S1 Fig Crystal structure ligand conformation was regenerated (RMSD = 0.3 Å, binding score = -25.79 kcal/mol and LEI = 1.5). (TIF)

S2 Fig Examples of potential *Mtb* targets predicted by MCNBC. A total of 25 proteins were assigned 132 compounds, each had Bayesian Score > = 1.0 and Z-score > = 1.5.
(TIF)

S3 Fig Examples of DHFR inhibitors. (TIF)

S1 Table Docking results showing the top 100 compounds. The compounds are listed in descending order of LEI.
(PDF)

S2 Table Predicted *Mtb* dihydrofolate reductase ligands predicted by MCNBN, SEA and Docking. (PDF)

## References

[1] WHO. Global Tuberculosis Control. 2011.

[2] Zignol M, van Gemert W, Falzon D, Sismanidis C, Glaziou P, et al Surveillance of anti-tuberculosis drug resistance in the world: an updated analysis, 2007–2010. Bull World Health Organ. 2012; 90: 111–119D. 10.2471/BLT.11.092585 22423162 PMC3302554

[3] Warner DF, Mizrahi V. Approaches to target identification and validation for tuberculosis drug discovery: A University of Cape Town perspective. South African Medical Journal. 2012; 102: 457–460. 22668936 10.7196/samj.5437

[4] WHO. Global Tuberculosis Report. 2013.

[5] Bajorath J. Integration of virtual and high-throughput screening. Nat Rev Drug Discov. 2002; 1: 882–894. 12415248 10.1038/nrd941

[6] Bento AP, Gaulton A, Hersey A, Bellis LJ, Chambers J, et al The ChEMBL bioactivity database: an update. Nucleic Acids Res. 2014; 42: D1083–1090. 10.1093/nar/gkt1031 24214965 PMC3965067

[7] Ballell L, Bates RH, Young RJ, Alvarez-Gomez D, Alvarez-Ruiz E, et al Fueling open-source drug discovery: 177 small-molecule leads against tuberculosis. ChemMedChem. 2013; 8: 313–321. 10.1002/cmdc.201200428 23307663 PMC3743164

[8] Lipinski CA Lead- and drug-like compounds: the rule-of-five revolution. Drug Discov Today Technol. 2004; 1: 337–341. 10.1016/j.ddtec.2004.11.007 24981612

[9] Florkowski CM Sensitivity, Specificity, Receiver-Operating Characteristic (ROC) Curves and Likelihood Ratios: Communicating the Performance of Diagnostic Tests. Clin Biochem Rev. 2008; 29: S83–S87. 18852864 PMC2556590

[10] Lipinski CA, Lombardo F, Dominy BW, Feeney PJ. Experimental and computational approaches to estimate solubility and permeability in drug discovery and development settings. Advanced Drug Discovery Reviews. 1997; 23: 3–25. 10.1016/s0169-409x(00)00129-0 11259830

[11] Yamanishi Y, Araki M, Gutteridge A, Honda W, Kanehisa M. Prediction of drug-target interaction networks from the integration of chemical and genomic spaces. Bioinformatics. 2008; 24: i232–i240. 10.1093/bioinformatics/btn162 18586719 PMC2718640

[12] Strombergsson H, Kleywegt GJ. A chemogenomics view on protein-ligand spaces. BMC Bioinformatics 10 2009; Suppl 6: S13 10.1186/1471-2105-10-S6-S13 19534738 PMC2697636

[13] Martı nez-Jime ´nez F, Papadatos G, Yang L, Wallace IM, Kumar V, Pieper U, et al Target Prediction for an Open Access Set of Compounds Active against Mycobacterium tuberculosis. PLoS Comput Biol. 2013; 9: e1003253 10.1371/journal.pcbi.1003253 24098102 PMC3789770

[14] Ekins S, Freundlich JS, Reynolds RC. Fusing dual-event data sets for Mycobacterium tuberculosis machine learning models and their evaluation. J Chem Inf Model. 2013; 53: 3054–3063. 10.1021/ci400480s 24144044 PMC3910492

[15] Kumar M, Vijayakrishnan R, Subba Rao G. In silico structure-based design of a novel class of potent and selective small peptide inhibitor of Mycobacterium tuberculosis Dihydrofolate reductase, a potential target for anti-TB drug discovery. Mol Divers. 2010; 14: 595–604. 10.1007/s11030-009-9172-6 19697148

[16] Dias MV, Tyrakis P, Domingues RR, Leme AF, Blundell TL. Mycobacterium tuberculosis Dihydrofolate Reductase Reveals Two Conformational States and a Possible Low Affinity Mechanism to Antifolate Drugs. Structure. 2014. 10.1016/j.str.2013.09.022 24210757

[17] Kappler MA. Software for rapid prototyping in the pharmaceutical and biotechnology industries. Curr Opin Drug Discov Devel. 2008; 11: 389–392. 18428093

[18] Gaulton A, Bellis LJ, Bento AP, Chambers J, Davies M, et al ChEMBL: a large-scale bioactivity database for drug discovery. Nucleic Acids Res. 2012; 40: D1100–1107. 10.1093/nar/gkr777 21948594 PMC3245175

[19] Rogers D, Hahn M. Extended-connectivity fingerprints. J Chem Inf Model. 2010; 50: 742–754. 10.1021/ci100050t 20426451

[20] Myint KZ, Wang L, Tong Q, Xie XQ. Molecular fingerprint-based artificial neural networks QSAR for ligand biological activity predictions. Mol Pharm. 2012; 9: 2912–2923. 22937990 10.1021/mp300237z PMC3462244

[21] Ekins S, Reynolds RC, Franzblau SG, Wan B, Freundlich JS, et al Enhancing hit identification in Mycobacterium tuberculosis drug discovery using validated dual-event Bayesian models. PLoS One. 2013; 8: e63240 10.1371/journal.pone.0063240 23667592 PMC3647004

[22] Nidhi, Glick M, Davies JW, Jenkins JL. Prediction of biological targets for compounds using multiple-category Bayesian models trained on chemogenomics databases. Journal of chemical information and modeling. 2006; 46: 1124–1133. 16711732 10.1021/ci060003g

[23] Xia X, Maliski EG, Gallant P, Rogers D. Classification of Kinase Inhibitors Using a Bayesian Model. J Med Chem. 2004; 47: 4463–4470. 15317458 10.1021/jm0303195

[24] Keiser MJ, Setola V, Irwin JJ, Laggner C, Abbas AI, et al Predicting new molecular targets for known drugs. Nature. 2009; 462: 175–181. 10.1038/nature08506 19881490 PMC2784146

[25] Lounkine E, Keiser MJ, Whitebread S, Mikhailov D, Hamon J, et al Large-scale prediction and testing of drug activity on side-effect targets. Nature. 2012; 486: 361–367. 10.1038/nature11159 22722194 PMC3383642

[26] Keiser MJ, Roth BL, Armbruster BN, Ernsberger P, Irwin JJ, et al Relating protein pharmacology by ligand chemistry. Nat Biotechnol. 2007; 25: 197–206. 17287757 10.1038/nbt1284

[27] Totrov Ma AR. Flexible Protein–Ligand Docking by Global Energy Optimization in Internal Coordinates. PROTEINS: Structure, Function, and Genetics Suppl. 1997; 1: 215–220. 9485515 10.1002/(sici)1097-0134(1997)1+<215::aid-prot29>3.3.co;2-i

[28] Abad-Zapatero C, Perisic O, Wass J, Bento AP, Overington J, et al Ligand efficiency indices for an effective mapping of chemico-biological space: the concept of an atlas-like representation. Drug Discov Today. 2010; 15: 804–811. 10.1016/j.drudis.2010.08.004 20727982

[29] Neves MA, Totrov M, Abagyan R. Docking and scoring with ICM: the benchmarking results and strategies for improvement. J Comput Aided Mol Des. 2012; 26: 675–686. 10.1007/s10822-012-9547-0 22569591 PMC3398187

[30] Abrahams K, Cox JAG, Spivey VL, Loman NJ, Pallen MJ, Constantinidou C, et al Identification of novel imidazo[1,2-a]pyridine inhibitors targeting M. tuberculosis QcrB. PLoS One. 2012; 7: e52951 10.1371/journal.pone.0052951 23300833 PMC3534098

[31] Rogers D, Brown RD, Hahn M. Using extended-connectivity fingerprints with Laplacian-modified Bayesian analysis in high-throughput screening follow-up. J Biomol Screen. 2005; 10: 682–686. 16170046 10.1177/1087057105281365

[32] Chen B, McConnell KJ, Wale N, Wild DJ, Gifford EM. Comparing Bioassay Response and Similarity Ensemble Approaches to Probing Protein Pharmacology. Bioinformatics. 2011; 27: 3044–3049. 10.1093/bioinformatics/btr506 21903625

[33] Blondeau JM. Fluoroquinolones: mechanism of action, classification, and development of resistance. Surv Ophthalmol. 2004; 49 Suppl 2: S73–78. 15028482 10.1016/j.survophthal.2004.01.005

[34] Mdluli K, Ma Z. Mycobacterium tuberculosis DNA Gyrase as a Target for Drug Discovery. Infectious Disorders-Drug Targets. 2007; 7: 1–10. 10.2174/187152607781001763 17970226

[35] Chen F, Mackey AJ, Stoeckert CJ Jr, Roos DS. OrthoMCL-DB: querying a comprehensive multi-species collection of ortholog groups. Nucleic Acids Res. 2006; 34: D363–368. 16381887 10.1093/nar/gkj123 PMC1347485

[36] Magarinos MP, Carmona SJ, Crowther GJ, Ralph SA, Roos DS, et al TDR Targets: a chemogenomics resource for neglected diseases. Nucleic Acids Res. 2012; 40: D1118–1127. 10.1093/nar/gkr1053 22116064 PMC3245062

[37] Li R, Sirawaraporn R, Chitnumsub P, Sirawaraporn W, Wooden J, Athappilly F., et al Three-dimensional structure of M. tuberculosis dihydrofolate reductase reveals opportunities for the design of novel tuberculosis drugs. Journal of molecular biology. 2000; 295: 307–323. 10623528 10.1006/jmbi.1999.3328

[38] Mdluli K, Kaneko T, Upton A. Tuberculosis drug discovery and emerging targets. Ann N Y Acad Sci: 1–20. 10.1111/nyas.12459 24920100

[39] Zheng J, Rubin EJ, Bifani P, Mathys V, Lim V, et al Para-Aminosalicylic acid is a prodrug targeting dihydrofolate reductase in Mycobacterium tuberculosis. J Biol Chem. 2013; 288: 23447–23456. 10.1074/jbc.M113.475798 23779105 PMC5395024

[40] Leduc D, Escartin F, Nijhout HF, Reed MC, Liebl U, Skouloubris S, et al Flavin-Dependent Thymidylate Synthase ThyX Activity: Implications for the Folate Cycle in Bacteria. J Bacteriol. 2007; 189: 8537–8545. 17890305 10.1128/JB.01380-07 PMC2168944

