# Supplementary figures and images for: Mycobacterial Dihydrofolate Reductase Inhibitors Identified Using Chemogenomic Methods and In Vitro Validation
Source: PLoS One. 2015 Mar 23;10(3):e0121492. doi: 10.1371/journal.pone.0121492 (PMC4370846; doi:10.1371/journal.pone.0121492)

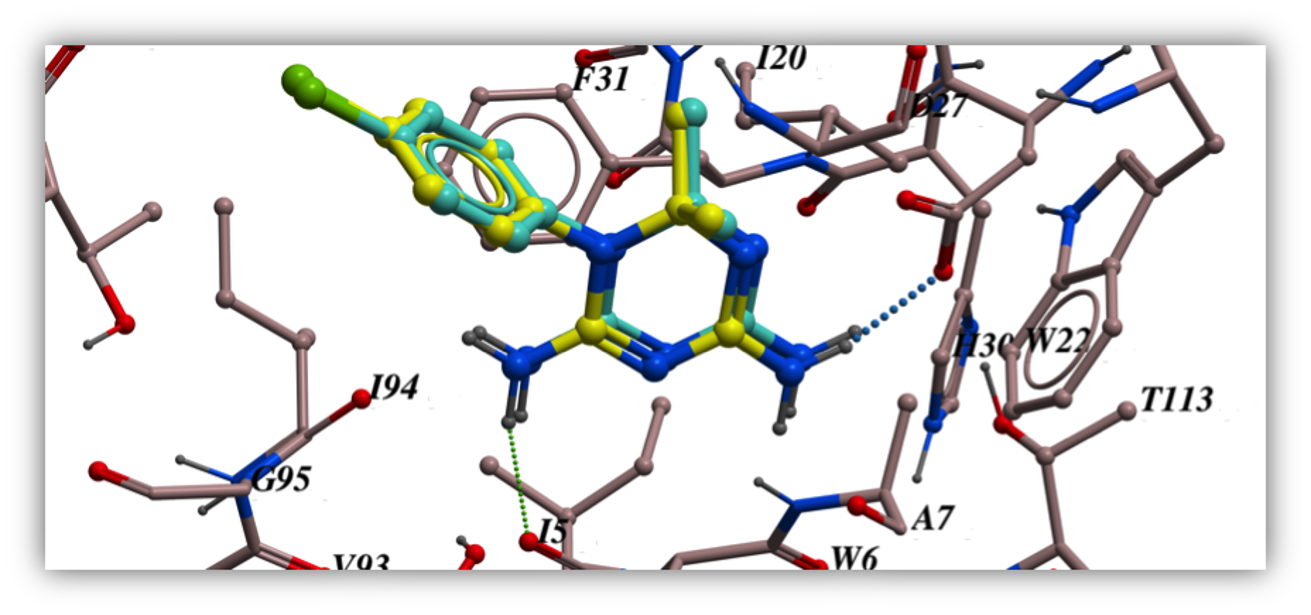

Supplement: S1 Fig — (TIF) [file pone.0121492.s001.tif]

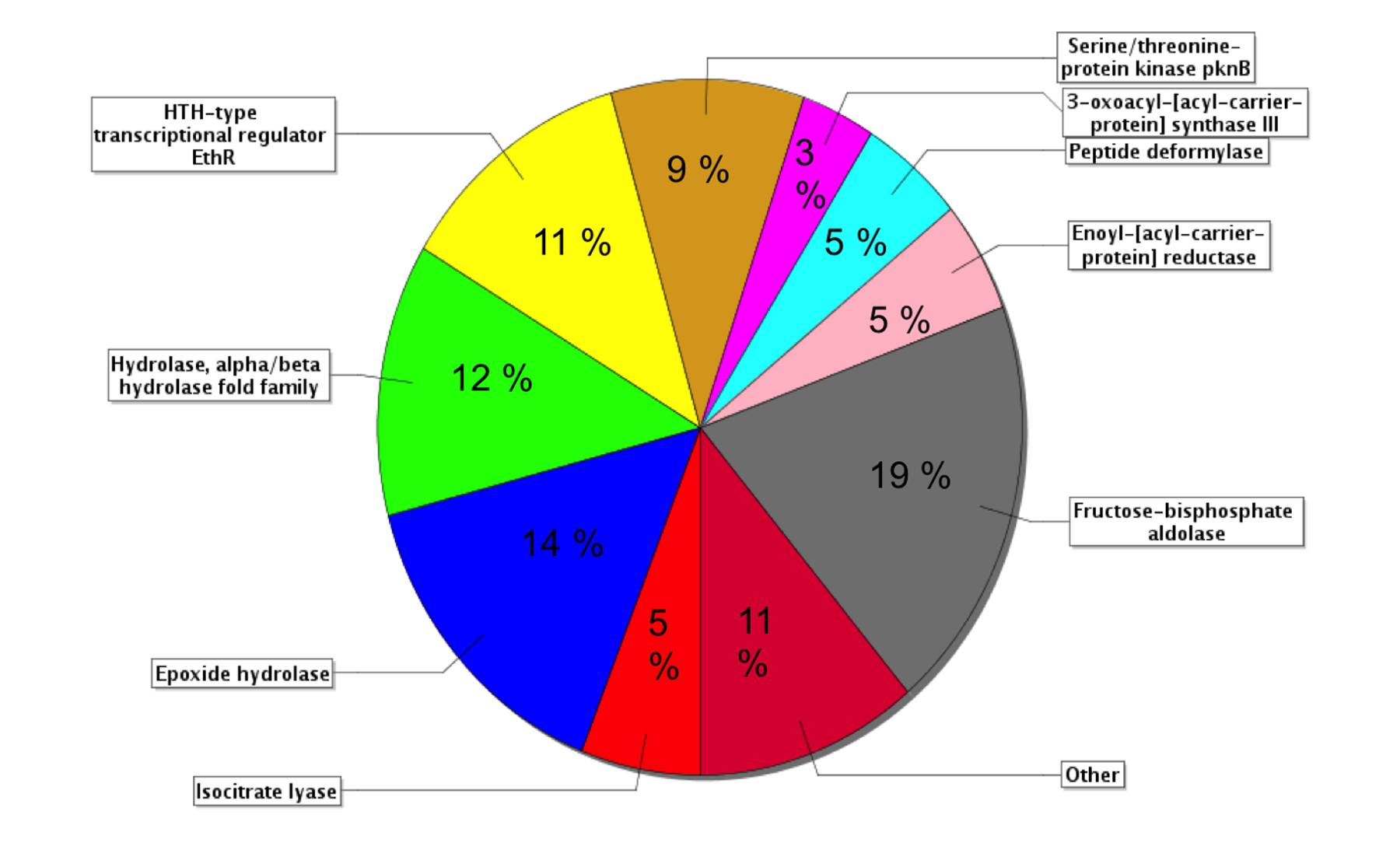

Supplement: S2 Fig — A total of 25 proteins were assigned 132 compounds, each had Bayesian Score > = 1.0 and Z-score > = 1.5. (TIF) [file pone.0121492.s002.tif]

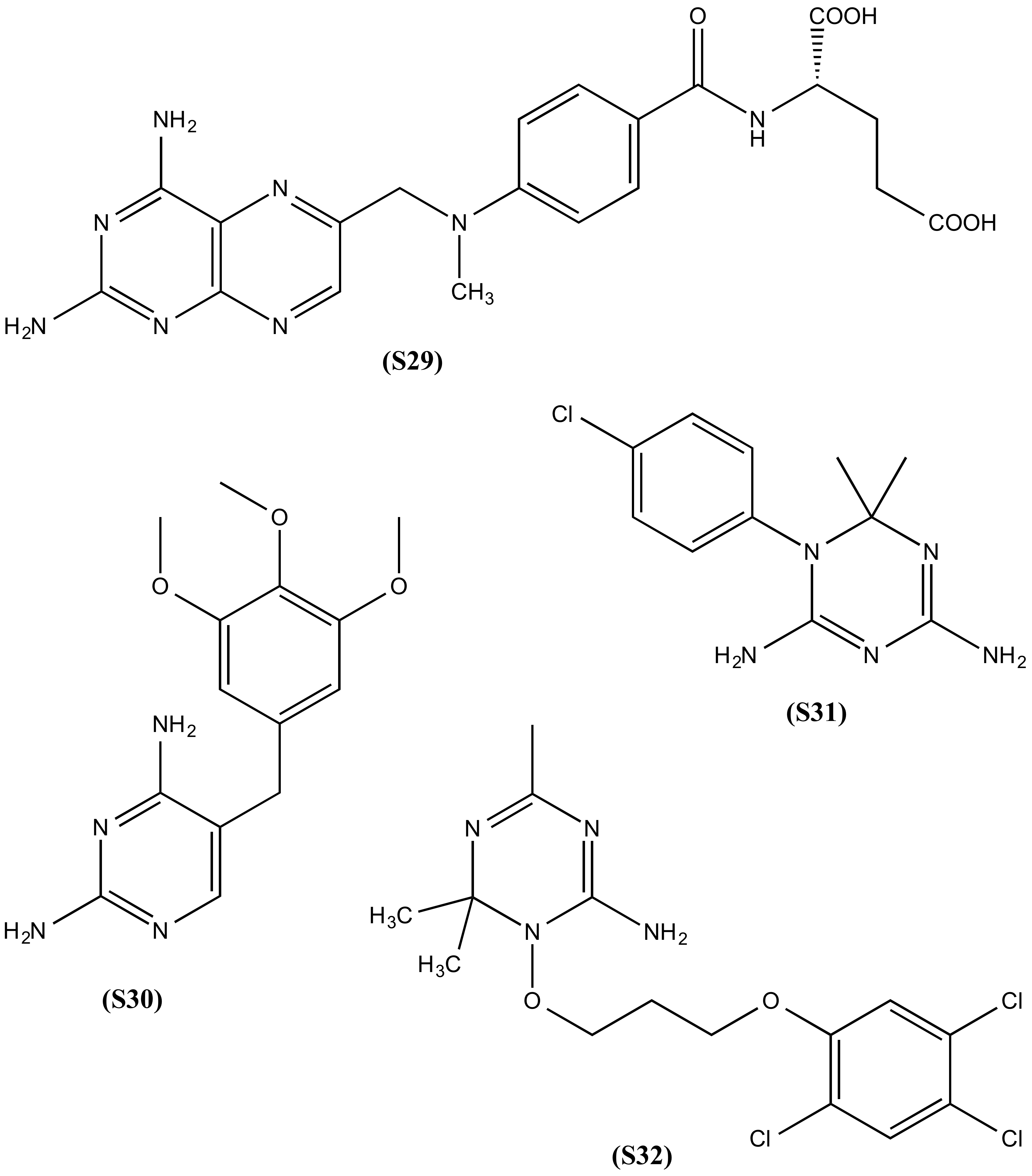

Supplement: S3 Fig — (TIF) [file pone.0121492.s003.tif]
