# Supplementary material for: Mycobacterial Dihydrofolate Reductase Inhibitors Identified Using Chemogenomic Methods and In Vitro Validation
Source: PLoS One. 2015 Mar 23;10(3):e0121492. doi: 10.1371/journal.pone.0121492 (PMC4370846; doi:10.1371/journal.pone.0121492)
Supplement: S1 Table — The compounds are listed in descending order of LEI. (PDF) [file pone.0121492.s004.pdf]

Table S1: Docking results showing the top 100 compounds. The compounds are listed in descending order of LEI.

Compound Name	Molecular weight (g/mol)	No. of heavy atoms	ICM score (kcal/mol)	Ligand efficiency Index	Polar surface area	aLogP	No. of H-bond acceptors	No. of H-bond donors
Sb-439950	248.71	17	-24.60	1.45	47.28	3.28	3	2
gsk1365028a	339.41	24	-34.55	1.44	89.79	3.63	3	1
gsk747165a	214.26	16	-22.48	1.41	47.28	2.62	3	2
Brl-51091am	360.24	23	-31.63	1.38	95.96	1.61	6	3
gr119270b	221.23	16	-20.93	1.31	84.9	0.75	2	3
gw369335x	318.40	23	-29.80	1.30	103.11	3.98	5	2
Brl-51100am	341.41	25	-31.92	1.28	95.96	1.19	6	3
Brl-51093am	319.40	23	-29.26	1.27	95.96	1.25	6	3
Brl-8903sa	305.38	22	-27.59	1.25	95.96	0.76	6	3
gr135486x	219.67	15	-18.59	1.24	50.94	2.69	3	2
Brl-10143sa	319.40	23	-28.40	1.23	95.96	1.22	6	3
Brl-8088sa	311.38	23	-27.71	1.20	89.22	2.04	6	2
gr201245x	281.40	21	-24.33	1.16	41.29	2.59	3	2
Sb-381759	230.24	17	-19.61	1.15	60.44	-0.15	3	0
Skf-18326	227.24	17	-19.48	1.15	49.36	1.55	3	0
gw324595a	302.80	21	-23.98	1.14	45.11	3.91	2	2
gi105268x	252.20	18	-20.12	1.12	120.44	-3.20	6	2
gsk1839228a	430.54	32	-35.00	1.09	64.78	3.90	4	1
gr135487x	253.22	18	-19.38	1.08	50.94	2.96	3	2
Brl-7940sa	347.46	25	-26.49	1.06	95.96	1.68	6	3
Sb-829405	384.88	27	-28.47	1.05	52.6	3.73	4	0
gsk426083a	326.82	20	-20.92	1.05	111.73	2.88	3	2
Brl-10988sa	375.47	27	-28.02	1.04	113.03	1.42	7	3
gsk1281439a	416.95	27	-27.70	1.03	124.25	3.98	4	1
gsk1758774a	412.32	24	-24.09	1.00	63.82	3.21	2	1
Sb-435634	228.25	17	-16.89	0.99	56.51	2.40	4	2
gw335118x	240.26	18	-17.70	0.98	86.18	1.63	4	2
gsk1900293a	324.21	19	-18.63	0.98	45.4	3.43	2	2
Sb-706404	351.26	20	-19.18	0.96	64.06	2.84	3	1
gsk1893692a	310.76	20	-18.99	0.95	95.66	2.32	3	2
gsk441195a	355.21	20	-18.91	0.95	95.66	2.41	3	2
gsk3011724a	267.35	18	-16.88	0.94	72.37	1.92	3	1
gsk353496a	211.31	13	-12.17	0.94	91.35	2.75	4	1
gsk1213629a	358.44	24	-22.34	0.93	130.54	2.83	6	1
gsk1568930a	311.34	23	-21.35	0.93	81.16	3.25	3	1
Sb-657505	310.78	22	-20.37	0.93	44.29	5.35	3	3

gsk1783682a	377.40	28	-25.87	0.92	101.52	3.42	4	2
gsk1600166a	263.32	18	-16.62	0.92	98.39	1.59	4	2
gsk160672a	384.88	27	-24.91	0.92	61.38	3.26	4	1
gsk676381a	352.41	25	-22.93	0.92	95.66	3.39	3	2
gsk468214a	389.46	26	-23.76	0.91	179.13	1.91	7	2
gv187303x	290.36	22	-20.07	0.91	47.28	4.20	3	2
ah24048x	212.59	14	-12.77	0.91	72.63	1.38	4	1
Sb-830656	318.39	23	-20.92	0.91	61.83	1.93	5	0
gsk1801530a	313.78	22	-19.81	0.90	46.92	3.93	2	1
Sb-252384-a	387.27	24	-21.46	0.89	41.57	3.57	3	1
gsk260308a	321.31	22	-19.66	0.89	118.56	2.37	6	2
gsk731389a	335.44	22	-19.50	0.89	108.86	2.57	5	0
gsk1447646a	447.57	31	-27.45	0.89	118.78	4.60	3	1
gsk957094a	265.31	20	-17.65	0.88	46.4	2.07	2	1
gsk1829660a	307.39	23	-20.25	0.88	46.4	3.47	2	1
gsk1761215a	313.33	22	-19.37	0.88	117.15	1.83	4	2
gsk1926119a	338.38	24	-21.03	0.88	95.66	3.39	3	2
gsk281210a	380.21	22	-19.26	0.88	81.18	3.27	5	0
Sb-248988	365.25	24	-20.88	0.87	41.57	4.08	3	1
gsk798463a	335.36	25	-21.74	0.87	65.38	3.64	4	1
gsk409004a	335.42	24	-20.86	0.87	83.12	4.35	3	1
gsk1783646a	353.38	26	-22.58	0.87	97.61	2.17	5	2
gsk425180a	295.34	22	-19.00	0.86	75.86	3.82	5	2
Sb-254878	279.13	16	-13.81	0.86	37.37	3.16	2	1
gsk892651a	271.34	19	-16.39	0.86	74.63	1.81	2	1
gsk1238596a	369.48	26	-22.41	0.86	90.77	2.52	4	1
gr51104x	206.17	15	-12.92	0.86	50.69	1.38	3	1
gsk1303831a	328.34	23	-19.73	0.86	109.66	3.03	5	1
gsk2111534a	299.75	21	-18.00	0.86	46.4	2.74	2	1
Sb-830300	370.85	26	-22.27	0.86	52.6	3.15	4	0
gsk347301a	325.28	24	-20.40	0.85	117.16	2.19	4	2
gsk345724a	256.22	18	-15.25	0.85	48.14	3.11	3	1
gsk1752826a	380.34	26	-21.99	0.85	99.58	3.97	2	2
gsk124576a	385.25	24	-20.28	0.84	51.22	3.67	3	1
gsk1379183a	382.82	27	-22.78	0.84	72.19	4.49	2	2
Sb-729215-a	242.32	18	-15.18	0.84	34.15	1.86	3	1
Sb-615575	266.30	20	-16.78	0.84	63.83	3.47	4	1
gw360240x	324.38	24	-20.13	0.84	103.18	1.50	7	2
gsk1752829a	316.37	22	-18.31	0.83	95.66	2.72	3	2
gsk490439a	353.41	26	-21.46	0.83	54.04	2.47	4	1
gsk1761037a	314.36	22	-18.13	0.82	99.58	2.49	2	2
gsk1296323a	364.25	23	-18.76	0.82	83.12	4.20	3	1
gsk1658592a	371.77	26	-21.19	0.82	98.74	1.59	4	3
Sb-255019-m	374.86	26	-21.19	0.81	59.59	3.59	4	2
gsk921190a	327.42	22	-17.88	0.81	108.42	3.38	4	1
gsk686665a	365.47	27	-21.86	0.81	70.22	3.11	3	3

gsk1825341a	397.45	28	-22.60	0.81	125.1	1.66	5	2
gsk920656a	325.34	23	-18.56	0.81	101.58	2.60	5	1
Sb-281244	306.36	23	-18.51	0.80	57.26	2.87	3	2
gsk854007a	389.88	25	-20.03	0.80	137.14	3.37	5	1
Sb-388225-a	372.85	26	-20.80	0.80	67.43	2.96	4	2
gsk1306952a	329.80	22	-17.59	0.80	83.12	3.54	3	1
gsk273492a	359.81	26	-20.77	0.80	54.88	4.80	3	1
gsk1826247a	465.54	33	-26.35	0.80	97.29	3.49	6	2
Brl-18223av	257.29	19	-15.07	0.79	66.47	2.40	4	2
gsk1829820a	285.36	20	-15.85	0.79	74.63	2.30	2	1
gsk515917a	362.86	23	-18.19	0.79	121.31	3.70	5	1
gw560770x	241.27	18	-14.16	0.79	78.17	1.28	4	2
gsk1022128a	385.46	27	-21.21	0.79	105.48	3.34	4	2
gsk975842a	361.32	26	-20.40	0.78	68.02	4.37	3	1
gsk1826248a	478.57	34	-26.66	0.78	119.41	2.66	8	2
gsk463114a	336.38	25	-19.59	0.78	60.45	2.91	4	1
gr118224a	320.81	22	-17.15	0.78	34.47	3.54	3	0
gsk787059a	379.20	25	-19.47	0.78	90.14	3.18	5	1
