# Supplementary material for: Mycobacterial Dihydrofolate Reductase Inhibitors Identified Using Chemogenomic Methods and In Vitro Validation
Source: PLoS One. 2015 Mar 23;10(3):e0121492. doi: 10.1371/journal.pone.0121492 (PMC4370846; doi:10.1371/journal.pone.0121492)
Supplement: S2 Table — (PDF) [file pone.0121492.s005.pdf]

Table S2: Predicted *Mtb* dihydrofolate reductase ligands predicted by MCNBN, SEA and Docking

No.	Structure	Molecular weight (g/mol)	MCNBC Z-score	SEA E-value	Docking LEI
**S1**	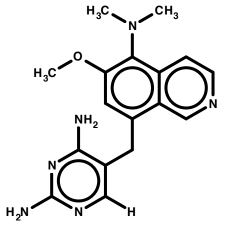 GW360240X	324.38	6.93	5.93E-97	0.84
**S2**	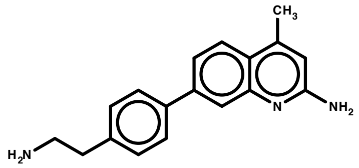 GW351921X	277.36	3.14	2.38E-14	0.74
**S3**	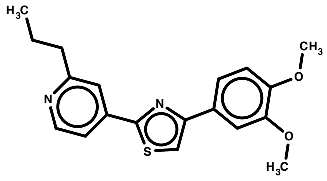 GSK1272261A	340.44	2.86	n.d	0.40
**S4**	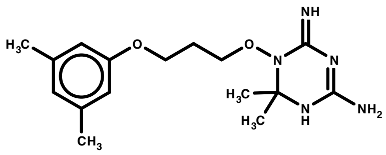 BRL-51093AM	319.40	2.78	1.55E-88	1.27
**S5**	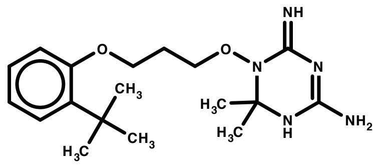 BRL-7940SA	347.46	2.21	1.01E-55	1.06
**S6**	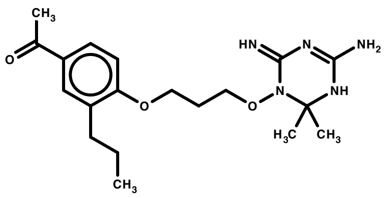 BRL-10988SA	375.47	2.20	1.02E-07	1.04

**S7**	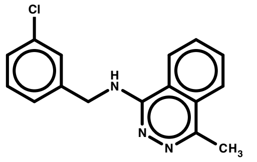 GSK1302651A	283.76	2.20	n.d	0.64
**S8**	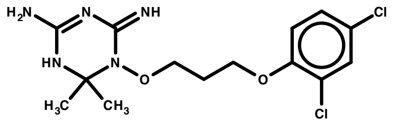 BRL-51091AM	360.24	2.02	1.99E-50	1.38
**S9**	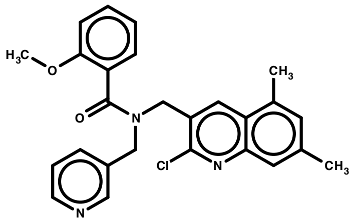 GSK497430A	445.94	2.02	n.d	0.38
**S10**	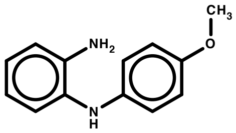 GSK747165A	214.26	2.01	n.d	1.41
**S11**	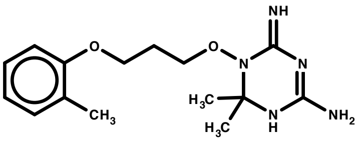 BRL-8903SA	305.38	1.93	2.66E-78	1.25
**S12**	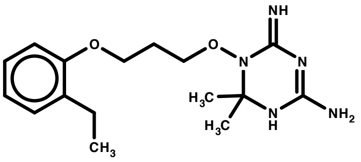 BRL-10143SA	319.40	1.90	2.39E-67	1.23
**S13**	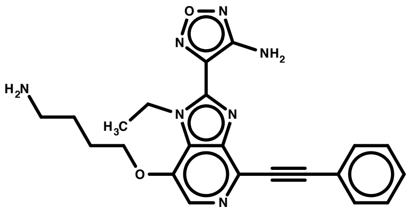 GSK460708A	417.46	1.88	n.d	0.59

**S14**	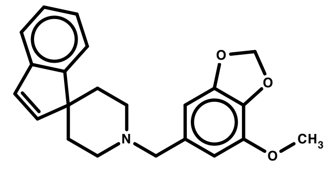 SB-354364	349.42	1.78	n.d	0.35
**S15**	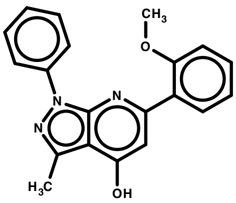 GSK1277359A	331.37	1.68	n.d	0.77
**S16**	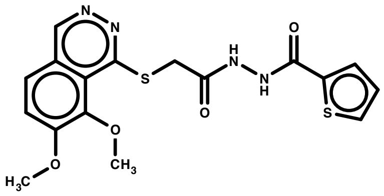 GSK359447A	404.46	1.66	n.d	0.74
**S17**	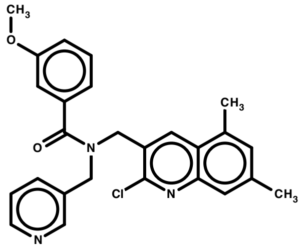 GSK497472A	445.94	1.65	n.d	0.47
**S18**	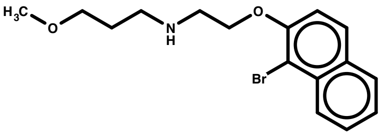 GSK152499A	338.24	1.58	n.d	0.56
**S19**	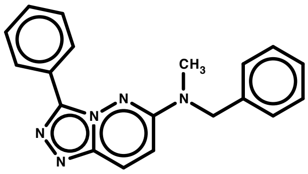 GSK870943A	315.37	1.58	n.d	0.67
**S20**	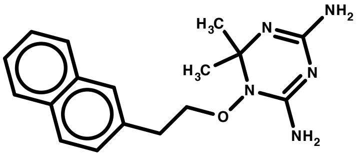 BRL-8088SA	311.38	2.10	3.27E-174	1.20

**S21**	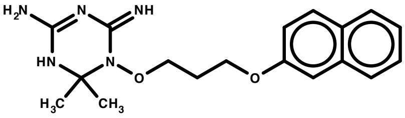 BRL-51100AM	341.41	2.19	1.62E-92	1.28
**S22**	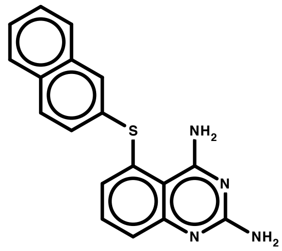 GW369335X	318.40	1.59	4.06E-82	1.30
**S23**	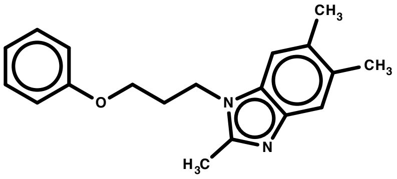 GSK1664204A	294.39	n.d	24E-10	n.d
**S24**	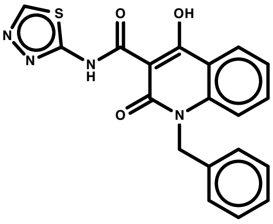 GSK391898A	378.40	n.d	8.81E-07	0.63
**S25**	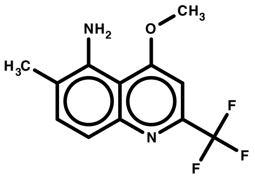 GSK345724A	256.22	1.64	1.29E-06	0.85
**S26**	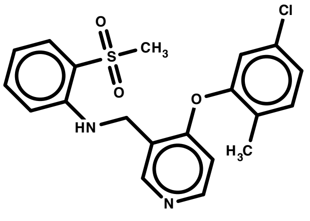 GSK1427115A	402.89	n.d	9.05E-06	0.60
**S27**	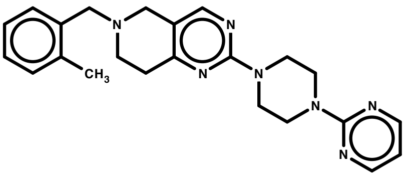 GSK1553808A	401.51	n.d	4.69E-04	0.31

**S28**	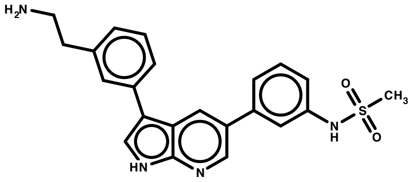<br>GSK581005A	406.50	n.d	2.32E-02	0.55
